# In vivo and in vitro evaluation of the effects of *Urtica dioica* and swimming activity on diabetic factors and pancreatic beta cells

**DOI:** 10.1186/s12906-016-1064-6

**Published:** 2016-03-15

**Authors:** Abbas Ranjbari, Mohammad Ali Azarbayjani, Ashril Yusof, Abdul Halim Mokhtar, Samad Akbarzadeh, Mohamed Yousif Ibrahim, Bahman Tarverdizadeh, Parviz Farzadinia, Reza Hajiaghaee, Firouzeh Dehghan

**Affiliations:** Department of Physical Education, Sanandaj Farhangyan University, Sanandaj, Iran; Exercise Physiology Department, Faculty of Physical Education, Islamic Azad University, Central Tehran Branch, Tehran, Iran; Department of Exercise Science, Sports Centre, University Malaya, 50603 Kuala Lumpur, Malaysia; Biochemistry Department, Faculty of Medicine, Bushehr University of Medical Sciences, Bushehr, Iran; Department of Pharmacy, Faculty of Medicine, University of Malaya, 50603 Kuala Lumpur, Malaysia; Exercise Physiology Department, Faculty of Physical Education, Islamic Azad University, Bushehr Branch, Bushehr, Iran; Biology and Anatomical Sciences, Faculty of Medicine, Bushehr University of Medical Sciences, Bushehr, Iran; Pharmacognosy and Pharmaceutic Department of Medicinal Plants Research Center, Institute of Medicinal Plants, ACECR, Karaj, Iran; Department of Sport Medicine, Faculty of Medicine, University of Malaya, 50603 Kuala Lumpur, Malaysia

**Keywords:** Diabetes, Urtica dioica, Insulin resistance, Cholesterol, TG, Pancreatic islet beta cells, Swimming exercise

## Abstract

**Background:**

*Urtica dioica* (UD) has been identified as a traditional herbal medicine. This study aimed to investigate the effect of UD extract and swimming activity on diabetic parameters through in vivo and in vitro experiments.

**Methods:**

Adult WKY male rats were randomly distributed in nine groups: intact control, diabetic control, diabetic + 625 mg/kg, 1.25 g/kg UD, diabetic + 100 mg/kg Metformin, diabetic + swimming, diabetic + swimming 625 mg/kg, 1.25 g/kg UD, and diabetic +100 mg/kg Metformin + swimming. The hearts of the animals were punctured, and blood samples were collected for biochemical analysis. The entire pancreas was exposed for histologic examination. The effect of UD on insulin secretion by RIN-5F cells in 6.25 or 12.5 mM glucose dose was examined. Glucose uptake by cultured L6 myotubes was determined.

**Results:**

The serum glucose concentration decreased, the insulin resistance and insulin sensitivity significantly increased in treated groups. These changes were more pronounced in the group that received UD extract and swimming training. Regeneration and less beta cell damage of Langerhans islets were observed in the treated groups. UD treatment increased insulin secretion in the RIN-5F cells and glucose uptake in the L6 myotubes cells.

**Conclusions:**

Swimming exercises accompanied by consuming UD aqueous extracts effectively improved diabetic parameters, repaired pancreatic tissues in streptozotocin-induced diabetics in vivo, and increased glucose uptake or insulin in UD-treated cells in vitro.

## Background

Diabetes mellitus is one of the most common metabolic diseases caused by high blood glucose and lack of insulin production or sensitivity, which influence body system functions [1]. Insulin resistance syndrome is one of the metabolic dysfunctions that play a crucial role in the pathogenesis of type 2 diabetes mellitus [2, 3]. Obesity and high triglyceride (TG) levels are dependent risk factors for insulin resistance syndrome [4, 5]. The increase in the number of people afflicted with diabetes over the past two decades can be due to lessened physical activity, poor dietary habits, overweight or obesity, and psychological stress [6, 7]. Diabetes is an epidemic disease, and over 5 % of the total population or an estimated 135 million people are infected. Hence, the World Health Organization estimated a rising prevalence of this silent disease. Approximately 285 million people worldwide were infected in 2010 [8], and this number would likely reach about 380 million by 2025 [7], and 439 million by 2035 (7.7 %) [9–11]. These predictions estimate a growing burden of diabetes particularly in developing countries [10]. This silent disease will become the strongest and deadliest leading cause of death in humans worldwide in the next 25 years [6]. Diabetes in populated countries such as India, China, and United States is rapidly increasing. In India, 30 million people were diagnosed as diabetics in 1995, and by 2025, this number is estimated to reach 70 million [11, 12].

Medicinal plants have been identified globally as biological source and have been investigated extensively as crude material for treating various disease conditions because of their effectiveness and economic values. Plant-derived medicines are safer to use than their synthetic alternatives, offering profound therapeutic benefits and affordable treatments. Currently, more than 30 % of medicines derived from natural sources are used in hospitals and clinics [13–15]. Despite their useful roles, most of the chemical medicines used in diabetes have damaging side effects [14, 16]; therefore, practitioners have considered changing to alternative natural plant therapy [17, 18]. *Urtica dioica* (UD) is one of natural plants used in traditional medicine [19, 20]. It has been used for homeopathy allergies, anemia, internal bleeding, kidney stones, burns, and diabetes [21]. Aside from its antihyperglycemic, anti-proliferative [22], anti-oxidant [23], and anti-dandruff [24] properties, it has anti-inflammatory or antimicrobial activity and has been proven to cure infectious diseases [25]. Furthermore The effects of UD on glucose transporter-4 (GLUT4) translocation on L6 muscle cells show that this plant stimulates GLUT4 transport to plasma membrane and glucose uptake into skeletal muscle [26].

The chemical compounds of this plant are lectin, lecithin, potassium, calcium, acetophenone, acetylcholine, quercetin, quinic acid, chlorogenic acid, butyric acid, caffeic acid, carbonic acid, coumaric acid, formic acid, histamine, succinic acid, pantothenic acid, linolenic acids, palmitic acid, serotonin, stigmasterol, terpenes, choline, agglutinin, alkaloids, xanthophyll, chlorophyll, kaempferol, coproporphyrin, lignan, linoleic, and violaxanthin. UD also contains protein, fatty substance, albumins, carotene, vitamin C, oxalate, fixed oil in its seeds, provitamin A, vitamin B1, K, xanthophylls, silicium, ferric oxide, and sistosterin in its leaves [27–29]. Among the many different types of this plant, two-basis nettle (Urtica dioica L) has been known as a traditional medicine in the world [27]. Other positive effects of this plant are joint pain reduction, bone inflammation treatment [30], cure for urinary tract infectious diseases, coronary heart disease, diabetes, cancer, inflammation, psychotic disorders, liver inflammation, and viral and parasitic diseases [31, 32], as well as influences on physiological brain function with exercise [33].

Aerobic activity is one of the useful ways to cure or prevent diabetes [5, 7, 34]. Physical activity has associated effects on glycemic and lipid profiles as well as metabolic risk factors for cardiovascular diseases, such as reduction of insulin resistance, atherogenic lipid abnormalities, high blood pressure, and improvement of metabolic status [34, 35]. The effect of regular exercise on improving glucose metabolism is well known in type 2 diabetes [35, 36], which may be due, in part, to the muscle contraction with insulin-like action as well as training-induced adaptations [37, 38]. Both physical activity and UD extract consumption approaches result in hypoglycemia and hypolipidemia. Nevertheless, data on the effect of the combination of these two beneficial variables on diabetes are unavailable. To fill this research gap, the present study aims to investigate in vivo and in vitro evaluation of Urtica dioica effects and swimming activity on diabetic factors and pancreatic beta cells on streptozotocin diabetic with the use of synthetic metformin medicine and normal rats.

## Methods

### Collection and preparation of plant samples

UD leaves were collected before the flowering season from Saral mountainous rangelands of the Zagros Mountains in Tabriz Khatoon Village of Kordestan region (Iran). Samples were taken, identified, authenticated, and deposited at Herbarium of Biochemistry Department, university Malaya by agricultural expert with voucher specimen no (19–5792).

### Preparation of plant material aqueous extract

The UD leaves (6 kg) were rapidly washed, shade-dried for 7 days, and then grounded to powder using an electric mill. To prepare aqueous extract, 1000 g of powdered samples was infused using 90 % ethanol at a ratio of 1 to 10. The eluate solution was fully concentrated using rotary evaporator (K-1Karwerke, GMBH Sco KG, Germany, TYP: Rvo6-ML, 010388949) at 75 °C to omit the solution value. To prepare dry powder, the resulting material was put in the oven for four days at 37 °C. The final weight of the extracted matter was 60 g. The extract sample was mixed with distilled water and administered orally on daily basis doses of 1.25 and 0.625 (mg/kg/day) [39].

### Gas chromatography/mass spectrometry (GC-MS) analysis

**Fig. 1:**
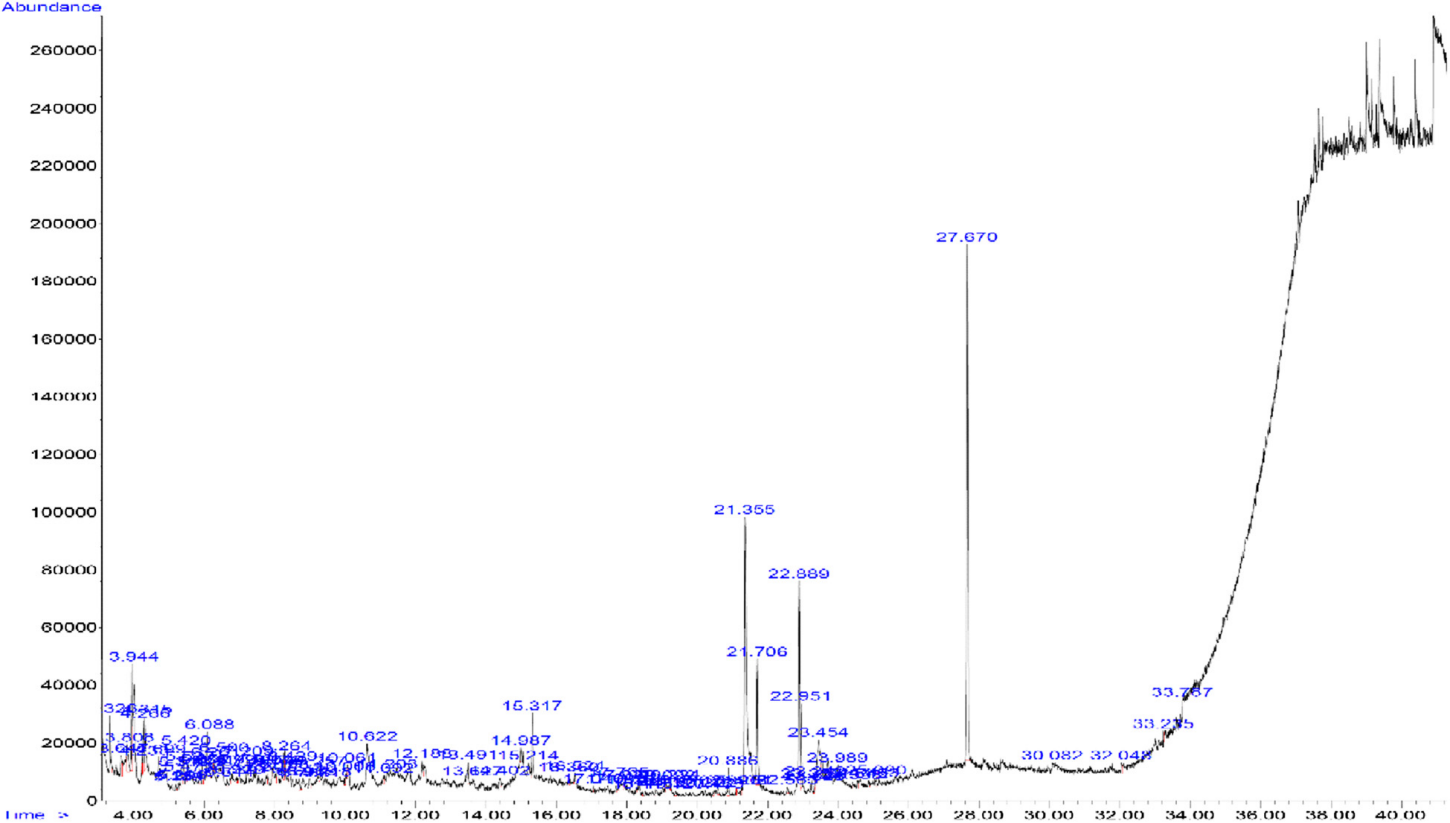
Chromatogram showing total ions of fiftheen herbicides and degradation products in standard solution

The chemical components of *UD* were determined by using the HPLC method. A 10 μl aliquot of filtrate sample was injected into LC-A6 (Shimadzu Co., Japan), equipped with a C18 column (Phenomenex Luna, 4.6 mm × 250 mm i.d., 5 μm) with mobile phase of water/acetonitrile 3/7 v/v. The main components of *UD* are presented in Table 1 and the chromatogram graphs of total ions in standard solution are shown in Fig. 1.

**Table 1:** Content of *UD* components in the mass spectra examined by HPLC

Peak #	RRt	Name of compound	%
1	3.82	Propylene Glycol	2.30
2	4.01	Diethylene Glycol = DEG = Digol	2.19
3	4.30	1, 8-Cineole = Eucalyptol	10.40
4	6.49	Ethyl Benzoate	2.96
5	14.35	Gamma-Dodecalactone = 4-octylbutane-4-olide	1.11
6	16.82	Di iso-Butyl Phthalate	3.01
7	17.94	Palmitic Acid	3.30
8	18.20	Dibenzosuberone	1.47
9	18.30	Ethyl Palmitate	1.39
10	18.61	4-methyl-2, 6-di-t-butyl Phenol = BHT	1.66
11	19.56	Methyl Oleate	2.04
12	20.22	Stearic acid	1.31
13	20.59	Ethyl Stearate	1.19
14	21.70	Tricosane	1.29
15	24.25	Di-(2-ethylhexyl) Phthalate = DEHP = DOP	40.07
			100

### In vitro studies

#### Cell culture

Rat pancreatic beta cell line (RIN-5F) and rat L6 myoblast cell line (L6) were used in this study. RIN-5 F and L6 cells were purchased from the American Type Culture Collection (ATCC, USA). L6 cells (ATCC, CRL-1458) were grown in Dulbecco’s Modified Eagle Medium (DMEM, Life Technologies, Inc., Rockville, MD, USA) and RIN-5 F (ATCC, CRL- 2058) cells were cultured in RPMI-1640 (Sigma–Aldrich, St. Louis, MO, USA). The cells were supplemented with 10 % fetal bovine serum (FBS, Sigma–Aldrich, St. Louis, MO, USA) and 1 % antibiotics (100 IU/mL of penicillin and 100 μg/mL of streptomycin (iDNA, South America) and were maintained in a humidified 5 % CO2 incubator at 37 °C. Cells were seeded in a flask at the required density per well and incubated for the desired time prior to the experiments.

#### Cell viability assay

The influence of UD extract on RIN-5F and L6 cells was evaluated by 3-(4, 5-dimethylthiazol-2-yl)-2, 5-diphenyltetrazolium bromide (MTT) assay. For this purpose, cells were seeded in a 96-well plate at density of 5 × 10^3^ cells/well with 1 mL of culture medium. After 24 h incubation at 37 °C in 5 % CO2, culture media were replaced by new media containing different concentrations (0, 0.375, 0.75, 1.5, 3, and 5 mg/mL) of UD extracts. UD extracts were prepared and transferred to the cells in the 96-well plate and incubated for an additional 48 h. After a specified period, the medium was discarded, and adherent cells were washed with phosphate buffer solution (PBS). About 20 μL of MTT solution (5 mg/mL MTT bromide in PBS) was added, and the mixture was incubated for 4 h at 37 °C. The medium was then removed, and the MTT formazan crystals formed by the metabolically viable cells were dissolved in 100 μL of dimethyl sulfoxide. Absorbance was measured at 595 nm. The assay was performed in triplicate.

#### Insulin secretion by the cultured RIN-5 F pancreatic cell

RIN-5F cells were seeded in 24-well plates at 2 × 10^5^ cells/well and incubated at 37 °C and 5 % CO2. After incubation for 24 h, the medium was removed from the wells, and the cells were washed twice with fresh medium containing low glucose (6.25 mM) or high glucose (12.5 mM). Afterward, the cells were incubated at 37 °C for 3 h with low glucose or high glucose medium, supplemented with 1 % FBS and treated with low or high concentrations of UD (1.5 and 3 mg/mL). Then, the aliquots in all wells were collected to determine the concentration of insulin in the media with the use of ELISA kit (Insulin ELISA kit, Ab100578, Abcam, Cambridge, UK) according to the manufacturer’s instructions. The insulin secretion levels at different concentrations of saffron were assessed by comparing them with the control insulin secretion level. The 0 concentration of extract (untreated cell) was considered as the control. The experiment was conducted in triplicate, and the data were presented as mean ± SD.

#### Determination of glucose uptake by cultured L6 myotubes

L6 myoblasts cells were subcultured in 24-well plates at 5 × 10^4^ cells/well and allowed to proliferate for 11 days to form myotubes in 0.4 mL of 10 % FBS/DMEM. The medium was refreshed every 3 days. The 11-day-old myotubes were kept for 2 h in Krebs–Henseleit buffer (pH 7.4, 0.141 g/L of MgSO4, 0.16 g/L of KH2PO4, 0.35 g/L of KCl, 6.9 g/L of NaCl, 0.373 g/L of CaCl2-2H2O, and 2.1 g/L of NaHCO3) containing 0.1 % bovine serum albumin, 10 mM Hepes, and 2 mM sodium pyruvate (KHH buffer). The myotubes were thereafter cultured in KHH buffer containing glucose (normal: 11 mM; high glucose: 25 mM) without or with UD extract (0, 1.5, and 3 mg/mL) for another 4 h. Glucose concentrations in the KHH buffer were determined with a glucose assay kit and a microplate reader (Appliskan, Thermo Fisher Scientific Inc., Waltham, MA, USA) at 508 nm, and the consumed glucose levels were derived from the differences in glucose concentrations between, before, and after culturing [40].

### In vivo studies

#### Animal

A total of 56 adult WKY (Wistar Kyoto) male rats (8–10 weeks of age, 253 ± 16 g of weight) were obtained from the Animal Center, Bushehr University of Medical Sciences and were caged under well ventilation in a standard environment (12 h light:dark cycle). The animals had free access to soy-free diet (Gold Coin Pellet) and tap water ad libitum. All procedures involving animal experiments were approved and conducted in strict accordance with the United States Institute of Animal Research guidelines for the care and use of laboratory animals [41] and approved by the Animal Care and Use Committee University Malaya Institutional with ethics number FIS/22/11/2011/FD(R). Blood sampling was performed at a specified time (8:10 a.m.) when the rats had had fasted at least 12 h. The animals were randomly divided into nine groups: intact control (C), diabetic control (CD), diabetic + 625 mg/kg UD (CD+ 625 UD), diabetic + 1.25 g/kg UD (CD + 1.25 UD), diabetic + 100 mg/kg Metformin (CD + M), diabetic + swimming (CD + E), diabetic + swimming + 625 mg/kg UD (CD + E + 625 UD), diabetic + swimming + 1.25 g/kg UD (CD + E + 1.25 UD), and diabetic + 100 mg/kg Metformin + swimming (CD + E + M). Six rats were used in each group. The hearts of the animals were punctured [42] after 4 weeks of swimming and UD feeding process. Blood samples were collected to investigate insulin, lipid, and lipoprotein indices. Whole pancreas tissue was exposed for histologic examination.

## Induction of experimental diabetes

To induce diabetes in rats, Streptozotocin (STZ, Enzo Life Sciences) was used by intraperitoneal injection (50 mg/kg). Distilled normal physiologic saline was used to prepare the injection solution. Rats were fasted 14 h before injection. The control group received normal saline. Blood sample for glucose measurements was collected from tail vein. A glucometer (Bionine-GM300) was used to measure blood glucose. Hyperglycemic animals with fasting blood glucose of more than 250 mg/dl were considered as diabetic.

Insulin resistance was calculated by following formula [43]:

$$ \mathrm{Insulin}\ \mathrm{resistance} = \mathrm{fasting}\ \mathrm{insulin}\left(\upmu \mathrm{U}/\mathrm{mL}\right) \times \mathrm{fasting}\ \mathrm{glucose}\ \left(\mathrm{mg}/\mathrm{dl}\right)/405 $$

Serum insulin level was evaluated by ultra-sensitive rat insulin ELISA Kit (Alpco-US). Insulin sensitivity was calculated by following formula [44]:

$$ 1/\left[ \log\ \mathrm{fasting}\ \mathrm{insulin}\ \left(\mathrm{mU}/\mathrm{L}\right) + \log\ \mathrm{fasting}\ \mathrm{glucose}\ \left(\mathrm{mg}/\mathrm{dL}\right)\right] $$

The Friedewald equation method was used to measure low-density lipoprotein (LDL) cholesterol concentration [45]. All data analyses were conducted in triplicate.

### Swimming protocol instructions

A swimming program was considered an exercise activity model in this study. Swimming was conducted in a clear plastic tank (70 cm × 90 cm × 150 cm) containing 30 cm of water (28 ± 0.5 °C). Before the exercise program, the swimming groups were familiarized to swimming (5 min/day in the first 3 days and 10 min/day in the second 3 days) to reduce stress. The exercise program consisted of swimming five times per week with gradual increases up to 4 weeks. The first week was for 15 min to 20 min at a depth of 20 cm; the second week was for 20 min to 30 min at a depth of 30 cm; the third week was for 30 min to 40 min at a depth of 40 cm; and the fourth week was for 45 min at a depth of 50 cm. the intensity of the exercise was monitored by increasing time and depth of water in plastic tank [39].

### Histological experiment sample preparation

After 4 weeks of tests, the hearts of the animals were punctured, and their pancreas tissues were exposed for histological experiment. The tissue was cleaned, fixed in 10 % formalin, and then paraffin embedded for microscopic procedures. Histopathology test was performed in hematoxylin and eosin stained at 5 μm thickness (Leitz 1512, Germany). The cellularity evaluation of Langerhans islets was conducted by light microscope (BX41 Olympus).

### Statistical analysis

All data values were mean ± standard deviation. The obtained data in this study were analyzed using SPSS version 17 and described in terms of central tendency and dispersion. Analysis of variance was used to evaluate the differences between the mean values. Kolmogorov–Smirinov test showed that the data were normally distributed. *p* value less than 0.05 was considered statistically significant.

## Results

### Cell proliferation assay

To assess the non-cytotoxic concentration of UD, the viability of RIN-5F and L6 cells were evaluated at doses ranging between 0 and 5 mg/mL using MTT assay. Within the tested concentrations, UD showed negligible cytotoxicity at 3–5 mg/mL in both tested cell lines (data not shown), and concentrations up to 3 mg/mL of UD were used in subsequent experiments.

### Determination of insulin secretion by cultured RIN-5 F pancreatic cells

**Fig. 2:**
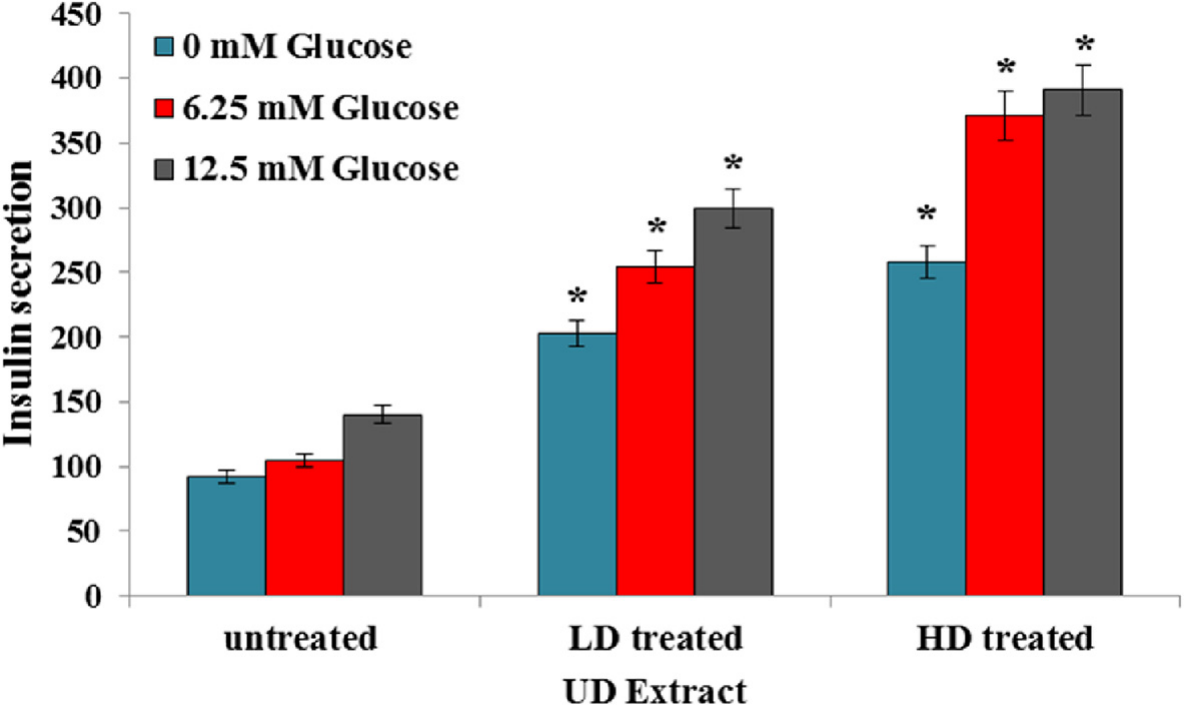
Effect of UD extract on glucose stimulated insulin release in RIN5 cells. Data were expressed as mean ± SD for 6 replicates. LD; low dose of UD-treated (1.5 mg/ml), HD; high dose of UD-treated (3 mg/ml). *P value less than 0.05 considered as significant comparing low dose and high dose of UD extract with untreated

As shown in Fig. 2, UD extract markedly increased the insulin secretion in both treated doses at glucose concentrations of 6.25 mm, and 12.5 mm significant induction of insulin secretion was observed in the RIN-5F cells treated with UD extract treatments.

### Glucose uptake by cultured L6 myotubes

**Fig. 3:**
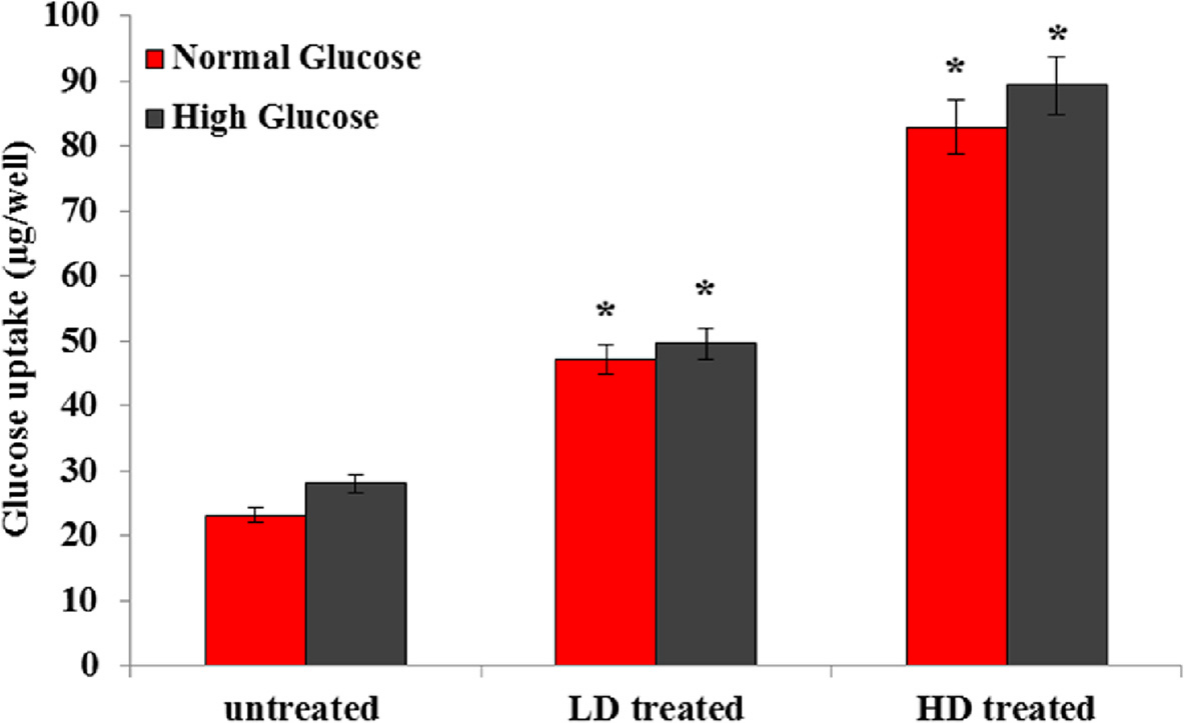
Effect of UD extract on glucose uptake in L6 myotubes. Data were expressed as mean ± SD for 6 replicates. LD; low dose of UD-treated (1.5 mg/ml), HD; high dose of UD-treated (3 mg/ml). *P value less than 0.05 considered as significant comparing low dose and high dose of UD extract with untreated

Differentiated L6 myotube cells were treated with UD to determine the function of UD in glucose metabolism of muscle cells, and their glucose uptakes were measured. As shown in Fig. 3, the glucose uptake of myotubes was considerably stimulated by the treatment of UD in a concentration-dependent manner at concentrations of 1.5 and 3 mg/mL under normal glucose (11.1 mM) and high glucose (25 mM) conditions. This result suggested that UD may act on proteins associated with glucose uptake signaling pathways in muscle cells.

### Biochemical parameter results

The effect of UD aqueous extract and exercise on weight loss in the control and administrated groups is shown in Table 2. Substantial weight loss was observed in diabetic groups. After 4 weeks of exercise activities, UD and metformin caused significant changes in the average weight. Weight gain was not significant in the treated groups; however, among treated diabetic groups, the group treated with UD dose of 1.25 g/kg with swimming activity had significant increase in weight compared with the diabetic control group (*p* < 0.05).

**Table 2:** Comparison of the effect of UD and exercise on changes in rat’s weight

	Intact control	Diabetic control	Diabetic + 625 mg/kg UD	Diabetic + 1.25 g/kg UD	Diabetic + Metformin	Diabetic + swimming	Diabetic + swimming + 625 mg/kg UD	Diabetic + swimming + 1.25 g/kg UD	Diabetic + Metformin + swimming
Week 1 day 7	83/256 ± 22/25	246 ± 64/17	14/217 ± 15/44	219 ± 02/32	5/225 ± 28/28	7/206 ± 15/38	20/207 ± 89/43	203 ± 60/34	70/223 ± 41/34
Week 2 day 14	17/261 ± 8/24	05/235 ± 99/15	57/216 ± 57/44	43/220 ± 77/31	228 ± 45/27	2/205 ± 53/37	4/209 ± 41/45	5/207 ± 1/34	228 ± 97/32
Week 13 day 21	2/271 ± 64/25	09/229 ± 86/13	79/217 ± 85/41	5/221 ± 61/34	8/231 ± 79/26	209 ± 83/37	4/215 ± 24/45	33/218 ± 51/33	6/234 ± 11/32
Week 4 day 28	52/276 ± 63/28	08/227 ± 29/14	79/220 ± 28/38	36/228 ± 07/35	98/237 ± 94/27	8/209 ± 21/37	4/223 ± 36/43	42/226 ± 97/34	7/241 ± 94/29

The results indicated that blood glucose in the treated groups had significant decrease compared with the diabetic control group (*p* < 0.05). Maximum reduction was observed in the diabetic group administrated with 1.25 g/kg UD with swimming activity compared with the diabetic control group (*p* < 0.000) and other groups (*p* < 0.031). The blood glucose levels of all groups are presented in Table 3.

**Table 3:** The glycemic index result of study groups

Groups	Glucose concentration	Insulin concentration	Insulin resistance	Insulin sensitivity
C	108.5 ± 24.46^a^	13.05 ± 3.04	3.36 ± 0.48^a^	0.31 ± 0.01^a^
CD	467.16 ± 109.94	11.4 ± 3.53	11.68 ± 2.63	0.27 ± 0.01
CD + 0.625 UD	145.42 ± 14.16^a^	12.92 ± 2.37^a^	4.46 ± 0.26^a^	0.3 ± 0.006^a^
CD + 1.25 UD	158.28 ± 55.42^a^	15.24 ± 4.04^a^	5.54 ± 1.1^a^	0.29 ± 0.01^a^
CD + M	151.4 ± 30.55^a^	10.61 ± 1.18	3.89 ± 0.54^a^	0.31 ± 0.01^a^
CD + E	130.6 ± 11.23^a^	11.34 ± 1.08	3.53 ± 0.17^a^	0.31 ± 0.004^a^
CD + 0.625UD + E	136.4 ± 23.41^a^	12.56 ± 1.61^a^	3.81 ± 0.51^a^	0.31 ± 0.01^a^
CD + 1.25UD + E	107.33 ± 23.44^b^	13.14 ± 3.83^a^	2.16 ± 0.43^b^	0.34 ± 0.02^b^
CD + M + E	136 ± 23.27^a^	10.9 ± 1.54	4.2 ± 0.47^a^	0.31 ± 0.01^a^

^a^Indicates a significant difference with diabetic control

^b^Indicates a significant difference with negative control, diabetic control, and between-group differences

The insulin resistance level was 11.68 in the diabetic control group and 3.36 in the negative control. Homeostasis Model Assessment-Insulin Resistance (HOMA.IR) significantly decreased in the diabetic-treated groups compared with diabetic control after 4 weeks of administration (*p* < 0.000). The HOMA.IR value in the diabetic group treated with UD 1.25 g/kg and swimming was 2.16 and was 3.53 for the diabetic group without UD treatment. These groups showed maximum decrease of insulin resistance (*p* < 0.000 and *p* < 0.013, respectively). The results of insulin resistance are shown in Table 3.

Swimming program in combination with aqueous extract of UD treatment and metformin consumption in diabetic groups caused an increase in insulin sensitivity, insulin serum level, and pancreatic function index compared with the diabetic control group (*p* < 0.000). Insulin sensitivity increase was more pronounced in both negative and diabetic control groups, which were treated with UD 1.25 g/kg alongside swimming. No significant difference in insulin serum concentration was observed in the study groups (*p* > 0.05). Furthermore, the pancreatic function index in the treated group showed a significant increase compared with the non-treated diabetic group (*p* < 0.00), whereas maximum increase was observed in the group treated with UD extract dose of 1.25 g/kg (*p* > 0.05). The glycemic index results are presented in Table 3.

The results in Table 4 show a significant decrease in TG concentration of treated group compared with the diabetic control group (*p* < 0.00), reaching a value close to the level of the control group (*p* < 0.05). Maximum reduction in TG levels was observed in diabetics swimming (CD + E) and diabetics swimming + UD 1.25 g/kg (CD + UD 1.25 + E) groups (i.e., 24 and 28 mg/dl, respectively) (*p* < 0.05).

**Table 4:** The Serum lipid and lipoprotein results of study groups

Group	Cholesterol mg/kg	TG mg/kg	LDL mg/kg	HDL mg/kg
C	83.5 ± 5.71	61.33 ± 10.93^a^	16.66 ± 11.34	53.43 ± 11.39
C D	89 ± 11.08	118 ± 47.67	14.66 ± 11.69	64.38 ± 20.16
CD + 0.625 UD	89.57 ± 14.15	38.71 ± 12.53^a^	29.42 ± 22.61	50.15 ± 19.09
CD + 1.25UD	80.14 ± 22.89	58.14 ± 29.12^a^	22.42 ± 11.78	45.9 ± 10.79
CD + M	83 ± 25.62	51 ± 20.21^a^	22.6 ± 22.72	49.45 ± 9.07
CD + E	44.6 ± 7.43^a^	24 ± 1.58^b^	13.2 ± 6.45	33.12 ± 8.32^a^
CD + 0.625UD + E	81.4 ± 17.81	34 ± 16.34^a^	18 ± 16.77	51.42 ± 18.3
CD + 1.25 UD + E	58.83 ± 12.02^a^	28 ± 9.71^b^	14.16 ± 11.25	45.06 ± 12.72
CD + M + E	81.6 ± 26.65	57 ± 13.72^a^	19 ± 14.66	50.5 ± 18.38

^a^Indicates a significant difference with diabetic control

^b^Indicates a significant difference with negative control, diabetic control, and between-group differences

A significant decrease in the cholesterol concentration of diabetic + swimming (CD + E) and diabetic + swimming + UD 1.25 g/kg (CD + UD 1.25 + E) groups was observed compared with the diabetic control (CD) and control groups (C) (*p* < 0.001). No significant changes were observed in the other treated groups (*p* > 0.05). The investigation of LDL and HDL levels showed that the 4-week exercise program, UD administration, and metformin had no significant effects on treated groups (*p* > 0.05). The results of serum lipid and lipoprotein are presented in Table 4.

### Histological results

**Fig. 4:**
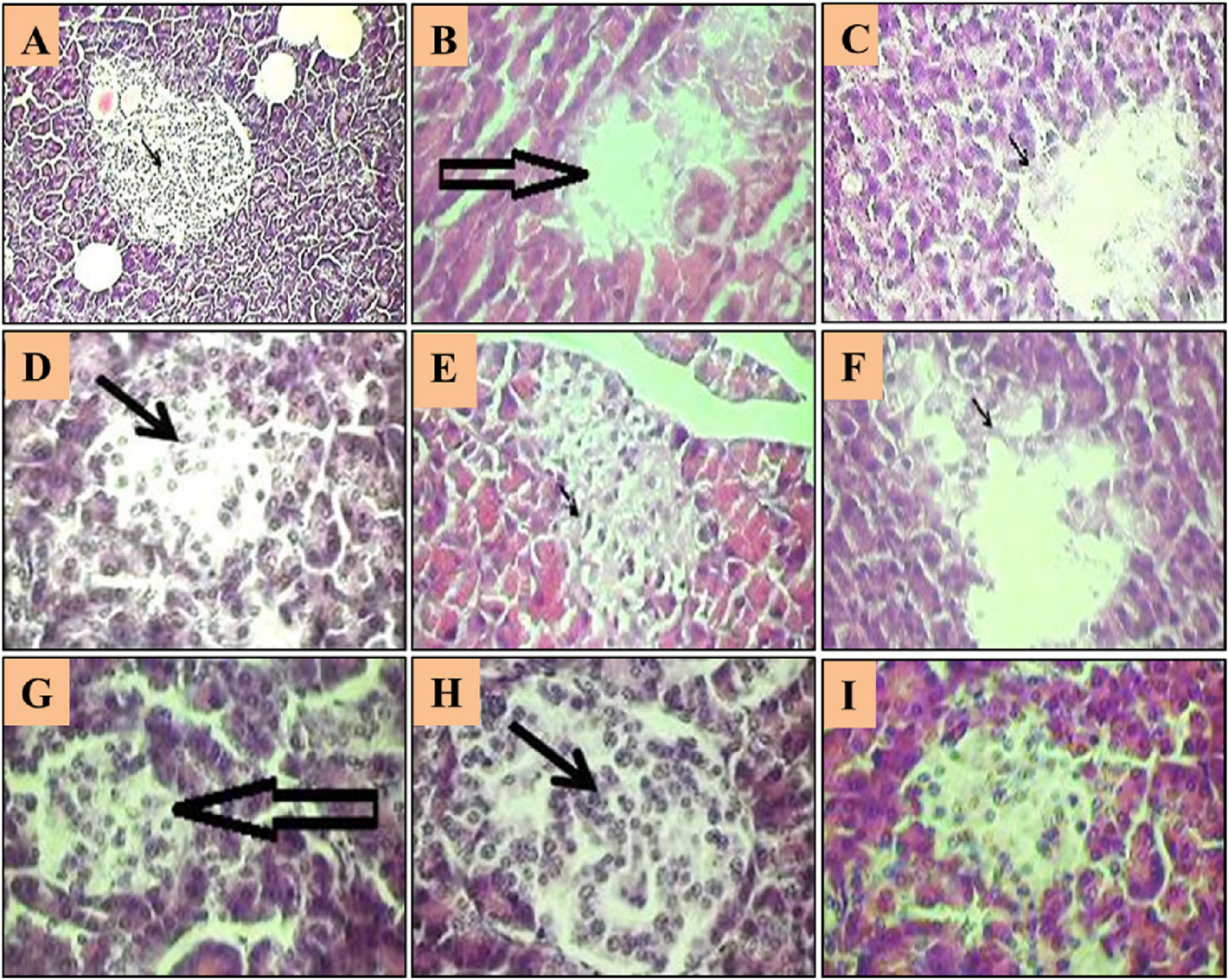
Comparison of the effect of UD extracts, swimming exercise and metformin treatment on the islets diameter in normal and diabetic rats; **a**: negative control; **b**: diabetic control; **c**: diabetic group with UD 0.625 mg/kg treated; **d**: diabetic group with UD 1.25 g/kg treated; **e**: diabetic group with metformin treated; **f**: diabetic group + exercise; **g**: diabetic group with 0.625 mg/kg treated + exercise; **h**: diabetic group with 1.25 g/kg treated + exercise; **i**: diabetic group with metformin treated + exercise (haematoxylin and eosin, original magnification × 400)

The administration of UD reduced the cellularity of the pancreatic islet beta cells compared with the diabetic control group. Significant increase in cellularity of islet and regeneration was observed in both groups, which received low dose and high dose of UD (Fig. 4a–d).

## Discussion

Our in vivo study results revealed that the 4-week administration of different doses of UD with exercises on diabetic rats caused significant decrease in diabetes markers, such as insulin resistance reduction, increased insulin sensitivity, lower TG and cholesterol, and improved function of the pancreatic beta cells compared with the diabetic group. Improved weight gain was noticed in the treatment groups. This positive change was more pronounced in the group that had swimming activity and consumed 1.25 g/kg UD. Increasing the enzyme hormone-sensitive lipase activity and stored triglycerides hydrolysis can release large amounts of fatty acids and glycerol into the blood circulation of patients with insulin deficient activity [46]. Thus, medications such as metformin that reduce blood triglycerides and cholesterol did not significantly change the lipid profiles, as observed in the present study. The data from the in vitro study indicated that UD extract increased insulin secretion through RIN-5F and glucose uptake by the L6 myotube cells.

The effects of UD on hypoglycemic, hypolipidemic, protection, and beta cell regeneration have been proven [15, 47] in several studies. Whereas Other studies have indicated that UD consumption has no effect on blood glucose and beta cell regeneration in diabetic rats [48]. By contrast, UD leaf extracts have been reported to have a protective role in increasing blood glucose and destroying pancreatic beta cells [19, 49]. Domola et al. [50] concluded that the compound Gazlin from UD extract has insulin-like effect in lowering blood glucose in diabetic patients. These differences in results may be caused by differences in regions, cities, and parts of UD (stalk, root, and leaves) used.

Our result supports the findings of Silevera et al. (2008), that is, blood sugar is reduced and weight is induced in diabetic rats with swimming activity [51]. The present data were consistent with the findings of Zinman et al. (2004) and confirmed the effect of exercise on blood sugar and insulin resistance [52]. Tjønna et al. [53] compared the effects of 90 % and 70 % aerobic exercise intensity on metabolic factors and insulin sensitivity in metabolic syndrome patients. Their data showed no significant differences in the weights of both exercise groups. Although insulin was increased, pancreatic beta-cell function had been observed by TG reduction and increased oxidation of free fatty acids. Nevertheless, pancreatic beta-cell function was significantly pronounced with exercise intensity. In this regard, aerobic capacity improvement, mitochondrial oxidation, and higher quality of life for individuals at high risk of diabetes were more pronounced in the periodic training group than in the continuous training group [54]. Although this study was conducted on humans, the results were consistent with the present study in terms of increase in insulin sensitivity, beta cell function, TG reduction, and fat metabolism.

Several studies have proven that moderate intensity exercise had no desirable effect on blood lipids, glucose, insulin, and insulin resistance [3, 5] The present study corroborates these findings. Aerobic activity has no significant effect on LDL and HDL but has a significant effect on blood glucose, cholesterol, TG reduction, insulin resistance, and insulin sensitivity.

A study conducted on diabetic rats by STZ and oral administration of aqueous extract of UD concluded that the aqueous extract of UD could have antihyperlipidemic and antihyperglycemic effects. Moreover, the aqueous extract of UD could decrease and increase glycemic markers and lipid levels [46], similar to the findings of this study. By contrast, Swanston Flatt showed that UD had no significant effect on blood glucose reduction in diabetic mice [19]. The effect of five natural plant extracts, including UD, on serum lipoprotein was investigated by Avci et al. They reported that the administration of UD on rats with full cholesterol consumption was closely associated with HDL enhancement and LDL abatement [55]. Furthermore, nettle seeds of Pilulifera reduced blood sugar and increased the number of Langerhans beta cells in diabetic rats [30].

The present study showed that the combination of aerobic exercises along with UD extract consumption could decrease blood sugar and insulin resistance, increase sensitivity of tissue to insulin, decrease fat serum, as well as improve the function and proliferation of Langerhans islets Beta cells, which were more pronounced in the group of UD consumption with swimming activities. As has been reported, UD extract can form a substance peptide loop (an amino acids chain) called Gazlin. Experiments conducted in this area have revealed that the attachment of 10 Gazlin molecules caused the formation of small tunnels on target tissues via cell membrane adhesion; this phenomenon made glucose to trickle into the cell, thereby reducing blood sugar [50]. It was also reported that the water extract of UD increased lipoprotein lipase (LPL) activity [46]. Thus, consumption of UD water extract and sport activities may decline the severity of diabetes. However, more studies are warranted to investigate the mechanisms of this plant.

## Conclusion

Use of simultaneous exercises and UD extract consumption can minimize diabetic markers, blood glucose, and weight gain and can increase insulin sensitivity, cellular fat metabolism, glucose carriers, insulin receptors in membrane, pancreatic beta cell proliferation, and glucose uptake stimulation and insulin secretion in vitro. Although the in vivo data suggested that UD and exercise training could cure diabetic rats, further investigation is needed to generalize these findings to human diabetes.
